# Nonsevere Hypoglycemia Episode Clinical and Economic Outcomes: A Comparison between Sulfonylurea and Sodium-Glucose Cotransporter 2 Inhibitor as Add-On to Metformin from a Canadian Perspective

**DOI:** 10.1155/2018/3718958

**Published:** 2018-07-08

**Authors:** Pendar Farahani

**Affiliations:** Department of Health Research Methods, Evidence, and Impact, McMaster University, Hamilton, ON, Canada

## Abstract

**Background:**

Nonsevere hypoglycemia episodes (NSHEs) are associated with clinically adverse outcomes, lower health-related quality of life, increased burden of disease, and reduced work productivity.

**Objective:**

To estimate prevalence of NSHEs and associated economic outcomes attributable to sulfonylurea (SU) versus sodium-glucose cotransporter 2 inhibitor (SGLT2i) initiation after metformin over one year for Canadian patients with type 2 diabetes (T2DM).

**Methods:**

Risk difference for NSHEs was calculated for SU and SGLT2i from RCT data. Estimation of NSHEs attributable to SU utilization in Canada was calculated from published data. Both direct and indirect costs associated with NSHEs were obtained from previous published studies in literature.

**Results:**

The number of patients with T2DM and exposure to SU in Canada in 2016 was estimated to be 1,246,438. The average underreported NSHEs in clinical settings were estimated at 67.7%. Risk difference for NSHEs for SU versus SGLT2i was estimated at 26.7%. Estimation of excess NSHEs attributable to SU utilization versus SGLT2i in Canada was estimated at 130,434 events per year (sensitivity analysis: minimum 80,680 and maximum 624,465). Total indirect costs including loss-of-work productivity and out-of-pocket costs secondary to excess NSHEs due to SU utilization versus SGLT2i after metformin were estimated at CDN$8.6M (M = millions) for 2016 (sensitivity analysis: minimum CDN$5.3M and maximum CDN$81.2M).

**Conclusion:**

NSHE, which is a forgotten variable in economic evaluations for healthcare reimbursement models, occurs frequently in real-world clinical settings but is infrequently reported. NSHEs can lead to a significant loss-of-work productivity and out-of-pocket costs.

## 1. Background

Both severe and nonsevere hypoglycemia are associated with clinical adverse outcomes, lower health-related quality of life, and increased burden of disease [1–4]. Nonsevere hypoglycemia episode (NSHE) reduces well-being and lowers quality of life by increasing anxiety and fear of repeated events, which can lead to negative lifestyle changes such as driving concerns and reduced work productivity [5, 6]. In general, severe hypoglycemia is defined as an episode of low blood glucose where a patient requires assistance of another person to actively administer carbohydrates, glucagon, or take other corrective actions; otherwise, an episode of low blood glucose can be categorized as nonsevere [7–9].

Currently, more than 10 classes of medication are available for diabetes pharmacotherapy [7–9]. Each class of medication has its own advantages and disadvantages from an efficacy and safety profile perspective [7–9]. In this milieu, enhanced individualized and patient-centred pharmacotherapy for diabetes is becoming more attainable than ever before [8]. Sulfonylureas (SUs) are associated with documented effective glucose-lowering outcomes, low cost, and decades of clinical experience in diabetes management [10]. However, SU usage is associated with risk of hypoglycemia, both severe and nonsevere [10]. On the other hand, newer classes of medications such as glucagon-like peptide 1 (GLP-1) receptor agonists and sodium-glucose cotransporter 2 inhibitor (SGLT2i) have safer profiles for hypoglycemia, can lead to weight loss, and lower high blood pressure [8]. Furthermore, recent studies illustrated that SGLT2i can reduce the risk of cardiovascular events in patients with diabetes [11–13]. Nonsevere hypoglycemia rate, attributable to SU utilization compared to newer classes such as SGLT2i, is of clinical significance [8].

The objective of this study was to estimate prevalence of nonsevere hypoglycemia episodes (NSHEs) and associated economic outcomes attributable to SU versus SGLT2i initiation after metformin over one year for Canadian patients with type 2 diabetes (DM2).

## 2. Methods

A search was conducted for RCTs of SGLT2i. The PubMed database was utilized for this search. The search date was set for published studies prior to January 15, 2016. The search was limited to RCTs reported in the English language for “canagliflozin,” “dapagliflozin,” and “empagliflozin.” RCTs selected had at least one arm of SU versus SGLT2i as an add-on to metformin [14]. The nonsevere hypoglycemia rates at 52 weeks were obtained for both the SU arm and the SGLT2i arm from studies that met the inclusion criteria (Table 1). Risk difference (RD) was calculated for the difference between the SU arm and the SGLT2i arm for NSHE rates at 52 weeks [14]. Of note, patient baseline characteristics including age, duration of diabetes, BMI, medication profile, and definition of NSHEs among selected RCTs were similar and comparable [14].

The following data were obtained from literature through the PubMed and Google Scholar databases: number of patients with DM2 in Canada [15] and utilizing medication [16]; pattern of SU utilization in Canada [17, 18]; probability of NSHEs for SU from RCTs and RWE studies [19–27]; and underreporting NSHEs in real-world clinical settings [5, 28–31]. Direct and indirect costs (including out-of-pocket costs and loss-of-work productivity) related to NSHEs per event were obtained from previously published literature (Table 2). All costs were adjusted according to Canadian dollars for 2016. Due to paucity of data from Canadian resources for direct and indirect costs of NSHEs, available data from European countries and the United States, where there are more similarities to the Canadian system, were incorporated.

Bibliography mining was also done on relevant articles to be as inclusive as possible. The primary estimation was conducted with the assumption of probability of only one episode of NSHE per annum per patient. However, the literature illustrated that compounded events of NSHEs per annum could occur [32–35], and therefore, costs as per compounded events of NSHEs per annum were also calculated.

## 3. Results

Type 2 diabetes accounts for 90% to 95% of the total cases of diabetes worldwide, and the number of patients with type 2 diabetes using medication is approximately 60% [16]. The total number of patients with diabetes in Canada is estimated to be around 3.5 million [15]; 51% are between 20 and 65 [36], and the percentage of type 2 diabetes on SU in Canada according to Canadian databases has been reported at 40% from the Canadian Primary Care Sentinel Surveillance Network (CPCSSN) database [17] and 37% from the Institute for Clinical Evaluative Sciences (ICES) database [18].

NSHEs frequently occur in real-world clinical settings but are infrequently reported. Average underreporting of NSHEs from literature [5, 28–31] was estimated to be 67.7% with a 95% confidence interval between 37.6% and 97.9%. On average, the probability of nonsevere hypoglycemia episodes from RCT data as a risk difference between SU and SGLT2i as an add-on to metformin is 26.7% with a standard deviation of 4.9% [19–24] (Table 1). The prevalence of NSHEs for patients with DM2 utilizing SU in real-world clinical settings has been reported between 10% and 30% [25–27].

With the assumption of probability of only one episode of NSHE per annum per patient, estimation of excess NSHEs attributable to SU utilization versus SGLT2i in Canada for 2016 was projected at 130,434 events per year (Table 3). With sensitivity analysis, estimated excess NSHEs were projected between 80,680 and 624,465 events per year (Table 3). Total indirect costs including loss-of-work productivity and out-of-pocket costs secondary to excess NSHEs due to SU utilization versus SGLT2i after metformin were estimated at CDN$8.6M for 2016 (Table 4). With sensitivity analysis, estimated excess costs were projected between CDN$5.3M and CDN$81.2M (Table 4).

Furthermore, frequency of events per patient per year for nonsevere hypoglycemia on SU in real-world studies has been reported with incidence between 2 and 12 events per patient per year [32–35]. With this consideration, average compounded events of NSHEs per annum could lead to 417,389 excess events per year attributable to SU utilization versus SGLT2i after metformin (Table 3). This can be translated to CDN$26.7M total indirect costs including loss-of-work productivity and out-of-pocket costs (Table 5).

## 4. Discussion

The current study illustrates the following findings: estimated NSHE incidence and prevalence in real-world clinical settings are high but are infrequently reported; the rate of NSHEs attributable to SGLT2i exposure is low in RCTs; the absolute risk difference for NSHE incidence is much higher for SU compared to SGLT2i after metformin in RCTs; estimated excess NSHEs attributable to SU utilization versus SGLT2i in Canada is substantial, which can lead to a significant increase in both direct and indirect costs and in particular loss-of-work productivity and out-of-pocket costs.

### 4.1. Nonsevere Hypoglycemia Episodes (NSHEs) and Pathophysiological/Clinical Adverse Outcomes

From a pathophysiological perspective, NSHE is associated with possible ischemic changes (T wave flattening), repolarization defects (increased QT intervals corrected for heart rate), and various cardiac arrhythmias [37]. These have been illustrated in a clinical study where patients with type 2 diabetes simultaneously utilized outpatient Holter monitors and continuous interstitial glucose monitors (CGM) [37]. From a clinical and outcome perspective, several RCTs, observational studies, and subsequent meta-analysis explored the relationship between hypoglycemia events and cardiovascular adverse outcomes [38, 39]. A population-based cohort study found dose–response relation between SU drugs and mortality in type 2 diabetes [40]. A meta-analysis illustrated a dose-dependent relationship between the severity of hypoglycemia and adverse vascular events and mortality [41]. Hazard ratio (HR) for mild hypoglycemia was 1.68 (*p* value less than 0.001), and HR for severe hypoglycemia was 2.33 (*p* value less than 0.001) [41]. In Canada, a large proportion of patients with T2DM who are utilizing SU are older than 65 and have significant cardiovascular risk factors or established atherosclerotic CV disease [17, 38]. The current study illustrates that SU utilization compared to SGLT2i after metformin may lead to more than 130,000 (400,000 compounded) excess NSHEs per annum for Canadian patients with type 2 diabetes.

### 4.2. Nonsevere Hypoglycemia Episodes (NSHEs) and Negative Impact on Quality of Life and Work Productivity

NSHEs impact quality of life and well-being. In the survey study from seven European countries, patients reported feeling tired, irritable, and having negative feelings following nonsevere hypoglycemia events [29]. After NSHEs, 59% of patients reported feeling tired or fatigued and 25% reported reduced alertness [31]. The negative effects on patients' emotional well-being lasted for 5 hours on average after NSHEs [31]. Nonsevere nocturnal hypoglycemic episodes lead to a greater disutility compared to nonsevere daytime episodes [4].

Subsequently, NSHEs impact work productivity and lead to increased costs of diabetes care. In the European survey, among respondents who were employed (48%), loss-of-work time after the last hypoglycemic event was reported for 9.7% of NSHEs. Overall, 10.2% (daytime) and 8% (nocturnal) of NSHEs led to work-time loss [29]. The mean of work-time loss was 84.3 minutes for daytime and 169.6 minutes for nocturnal NSHEs [29]. In another study, among employed patients, 9% of NSHEs led to an average lost work time of 1.4 hours in type 1 diabetes and 1.9 hours in type 2 diabetes per event [31]. In a US study [42], NSHEs reduced productivity with an average productivity loss of $2300 per person per year. After a nocturnal nonsevere hypoglycemia event, 23% of patients arrived late or missed work, 32% of patients missed a meeting or did not finish a task on time, and 15 hours of work was lost [42]. Work loss productivity is a very important aspect for patients with diabetes who work in Canada from a cost perspective, as Canadian average hourly earnings in 2016 for full-time employees were reported at $26.91 [43]. The current study illustrates that in Canada on average, more than CDN$6.8M (CDN$21.5M compounded) loss-of-work productivity due to excess NSHEs per annum may be attributed to SU utilization compared to SGLT2i after metformin.

### 4.3. Nonsevere Hypoglycemia Episodes (NSHEs) and Increase in Costs

Across European countries, there was a mean increase in blood glucose test use of three tests in the week following a NSHE [29]. In another report, in the week after a NSHE, blood glucose measurement increased by 8% in type 1 diabetes and 21% in type 2 diabetes [31]. In the US, NSHE increased treatment cost with blood glucose testing which went up by 5.6 extra tests within 7 days after a NSHE [42]. The current study illustrates that an estimated excess cost of CDN$1.4M (CDN$4.6M compounded) for direct costs and CDN$1.8M (CDN$5.2M compounded) for out-of-pocket costs can be attributed to SU utilization compared to SGLT2i after metformin due to excess NSHEs per annum for Canadian patients with type 2 diabetes.

### 4.4. Nonsevere Hypoglycemia Episodes (NSHEs) and Aging Population

The aging population is the most important demographic change affecting diabetes prevalence worldwide [44]. The aging of the Canadian population has been one of the factors contributing to the increase in the number of Canadians living with diagnosed diabetes [45–47]. In recent years, the highest increase in the number of individuals with diabetes in Canada was seen in the 60- to 64-year age group [45–47]. NSHEs are associated with significant chronic consequences leading to physical and cognitive dysfunction and eventually frailty and disability in elderly [48]. The incidence of hypoglycemia in older people (>75 years) with diabetes is difficult to estimate due to the limited number of clinical studies and the lack of standardization in hypoglycemia diagnosis [48]. In Canada, a large proportion of patients with DM2 who have exposure to SU are older than 65 and have significant cardiovascular risk factors or established atherosclerotic CV disease [17, 38]. This pattern of SU prescription for elderly in Canada can lead to a significant cost due to excess NSHEs per annum for elderly Canadian patients with type 2 diabetes [49].

### 4.5. Nonsevere Hypoglycemia Episodes (NSHEs) and High Prevalence of Occurrence in Real-World Clinical Settings

Several studies from Europe and North America reported that NSHEs frequently occur in real-world clinical settings but are infrequently reported to healthcare providers. A survey study from seven European countries reported that a high proportion of respondents rarely or never informed their general practitioner or specialist about hypoglycemia: 65% in type 1 diabetes and 50–59% in type 2 diabetes [30]. This study concluded that NSHEs are common among people with diabetes in real-world settings; however, many rarely or never inform their general practitioner or specialist about their hypoglycemia and the real burden of hypoglycemia may be underestimated [30]. In a US study, NSHEs were only reported by 25% of patients to a health care professional after an episode [42]. The current study illustrates that estimated NSHEs are high for Canadian patients with type 2 diabetes. Therefore, it is crucial to establish methods and processes to capture more NSHEs in real-world clinical settings in the face of the aging population and higher prevalence of diabetes in Canada [50].

### 4.6. Nonsevere Hypoglycemia Episodes (NSHEs) and Future Directions

Future clinical guidelines should be developed for comprehensive definition and reporting of hypoglycemia episodes, both severe and nonsevere, in RCTs and real-world clinical settings [51]. The initiatives are already underway with scientific organizations such as the Endocrine Society to address this issue [51]. Currently, there is a significant heterogeneity for definitions and reporting methods of hypoglycemia throughout RCTs and real-world clinical studies [52]. The implementation of a more comprehensive definition of hypoglycemia for all studies may result in less heterogeneity of the outcomes in evaluating safety and effectiveness of diabetes pharmacotherapy. Furthermore, measurement of blood glucose pattern with reliable, validated, high-tech methods such as continuous glucose-monitoring devices should be considered for all clinical studies [53].

Measurement of clinically important variables from patient-centred perspectives such as health-related quality of life (HRQoL) related to NSHEs should be implemented as part of all clinical studies [54]. Needs for development and validation of HRQoL tools for NSHE assessment along RCTs and real-world clinical studies should be addressed [55].

Despite the evidence, most economic models and guidelines do not capture and do not incorporate the impact of NSHEs on quality of life, work productivity, and related costs [56]. Furthermore, the cost-effectiveness evaluations in the current state can be very useful for policymakers but not for the individual patient level in a real-world clinical setting [57, 58]. Disutility scores are different for each individual patient, and utilizing the mean disutility score can be misleading at the individual patient level for tailored pharmacotherapy [57, 58]. Fortunately, in recent years, more guidelines are emphasizing on subgroup analysis in cost-effectiveness evaluation [59]. This facilitates subgroup-specific estimates of parameters in decision analytic models in cost-effectiveness analysis to compare different types of patients [60]. For example, in the case of the current study, the absolute annual acquisition cost difference between SU and SGLT2i was estimated to be CDN$1155 in favour of SU in Canada in 2016 [61]. However, with consideration of lost productivity and excess costs related to NSHEs for Canadian patients with employment, the recovery of SGLT2i acquisition costs due to reduced NSHE annual rate occurrences can be cost effective when there are at least three additional NSHE occurrences in a year with SU utilization compared with SGLT2i [62].

The limitations of this study are as follows: defining and reporting NSHEs were similar among studies; however, they were not exactly the same; lack of access to individual patient level data; for this study, only summary data was available through published studies, which by itself limits the ability to conduct further detailed analysis and data interpretation; literature for cost calculation were mainly from outside of Canada as there is a lack of data in literature for costs related to NSHEs from Canadian resources.

## Figures and Tables

**Table 1:** Extracted data from selected RCTs on SU versus SGLT2i as an add-on to metformin.

SGLT2i (+MET) versus SU (+MET)	Percentage of patients with NSHEs in SU arm (%)	Percentage of patients with NSHEs in SGLT2i arm (%)
Dapagliflozin versus glipizide [19]	39	3.4
Canagliflozin versus glimepiride [21]	31	5
Empagliflozin versus glimepiride [23]	20	1.5

**Table 2:** Costs associated with nonsevere hypoglycemic episodes (NSHEs) per episode from literature.

Direct costs: $11 per NSHE [63]	USD$ for 2015 [63]
Lost productivity: range from $15.26 to $93.47 per NSHE [42] Total out-of-pocket costs: $25.29 per NSHE [42]	USD$ for 2011 [42]
Total cost: $127 per person per event for nocturnal NSHE [64] Total cost: $70.67 per person per year for nocturnal NSHE [65]	USD$ for 2013 [64] CDN$ for 2013 [65]

**Table 3:** Estimation of excess NSHEs attributable to SU utilization versus SGLT2i after metformin in Canada for 2016.

	Base	Minimum	Maximum
With the assumption of probability of only one episode of NSHE per annum per patient	130,434 events per year	80,680	624,465
Estimated compounded events of NSHE per annum	417,389 events per year	260,868	5,047,762

Base scenario assumptions: average estimates for risk difference, number of patients with DM2 in Canada, pattern of SU utilization in Canada, and probability of NSHEs for SU; have not incorporated underreporting for NSHEs in real-world clinical settings. Minimum scenario assumptions: lower estimated boundaries for risk difference, number of patients with DM2 in Canada, pattern of SU utilization in Canada, and probability of NSHEs for SU; have not incorporated underreporting for NSHEs in real-world clinical settings. Maximum scenario assumptions: upper estimated boundaries for risk difference, number of patients with DM2 in Canada, pattern of SU utilization in Canada, and probability of NSHEs for SU; incorporated underreporting for NSHEs in real-world clinical settings.

**Table 4:** Estimated costs secondary to excess NSHEs due to SU utilization versus SGLT2i after metformin.

Scenarios (probability of single episode per patient per year)	Outcome in CDN$ for 2016		
	Base	Minimum	Maximum
Total indirect costs including work productivity and out of pocket	8.6M	5.3M	81.2M
Out-of-pocket costs	1.8M	1.1M	16.9M
Lost work productivity	6.8M	4.2M	64.3M
NSHE and self-treated (only direct cost)	1.4M	0.9M	6.9M

Base scenario assumptions: included only patients between 20 and 65; incorporated average income loss and costs; have not incorporated underreporting for NSHEs in real-world clinical settings. Minimum scenario assumptions: included only patients between 20 and 65; incorporated average minimum income loss and costs; have not incorporated underreporting for NSHEs in real-world clinical settings. Maximum scenario assumptions: included all patients; incorporated highest income loss and costs; incorporated underreporting for NSHEs in real-world clinical settings.

**Table 5:** Estimated costs secondary to excess NSHEs due to SU utilization versus SGLT2i after metformin.

Scenarios (compounded estimate)	Outcome in CDN$ for 2016
Total indirect costs including work productivity and out of pocket	26.7M
Out-of-pocket costs	5.2M
Lost productivity	21.5M
NSHE and self-treated (only direct cost)	4.6M

Scenario assumptions: included only patients between 20 and 65; incorporated average income loss and costs; have not incorporated underreporting for NSHEs in real-world clinical settings; average compounded incidence probability for NSHEs is 3 events per patient per year.
